# Large‐Scale Passive Acoustic Monitoring Data Shows Seasonal and Diel Diversity in Foraging Behaviour of Harbour Porpoises Within Their Distributional Range in the Northeast Atlantic

**DOI:** 10.1002/ece3.72566

**Published:** 2025-11-29

**Authors:** Jasmine Stavenow Jerremalm, Julie Beesau, Julia Carlström, Pia Eriksson, Mark Jessopp, Ailbhe Kavanagh, Olli Loisa, Mathilde Michel, Kylie Owen, Marianne Helene Rasmussen, Flore Samaran, Nicole Rose Eileen Todd, Emer Rogan

**Affiliations:** ^1^ School of Biological, Earth and Environmental Sciences (BEES) University College Cork Cork Ireland; ^2^ MaREI Centre, Sustainability Institute University College Cork Cork Ireland; ^3^ ENSTA, IPP, Lab‐STICC—UMR CNRS 6285 Brest France; ^4^ Department of Population Analysis and Monitoring Swedish Museum of Natural History Stockholm Sweden; ^5^ Marine Institute Oranmore Ireland; ^6^ Marine Environment Research Group Turku University of Applied Sciences Turku Finland; ^7^ Applied Ocean Physics and Engineering Department Woods Hole Oceanographic Institution Woods Hole Massachusetts USA; ^8^ The University of Icelands Research Center in Húsavík Húsavík Iceland

**Keywords:** behaviour, ecology, foraging, harbour porpoise, latitude, passive acoustic monitoring

## Abstract

Latitudinal gradient can influence ecosystem dynamics and species distribution, yet the influence on some aspects, such as intra‐species diversity, is less well understood. The large‐scale distribution of harbour porpoises ( *Phocoena phocoena* ) in the Northeast Atlantic, indicates that ecological adaptations within the species might be greater than currently recognised. This study investigates variation in foraging behaviour using long‐term passive acoustic monitoring data collected between 2009 and 2023 from Iceland, Sweden, Ireland, and France. In each area, Generalised Additive Models (GAMs) were used to investigate the influence of large‐scale environmental variables (water temperature, salinity, primary production, diel phase and daylength) on foraging behaviour. Our results show variability and complexity in foraging and differences in temporal patterns between areas. In the northernmost regions, with the largest variation in daylight, foraging behaviour was related to diel phase, with primarily nocturnal foraging recorded during the year, but with predominantly diurnal foraging in Iceland during late fall. In the southernmost regions, less effect of diel phase on foraging was found. Harbour porpoises in Sweden and Iceland exhibited increased day‐round foraging during calving periods, highlighting the role of reproductive energetics in behavioural adaptations, and the complexity and importance of foraging during different seasons. Our findings underscore the influence of environmental drivers in shaping foraging strategies, supporting the concept that harbour porpoises optimise these based on local conditions and prey availability. Using long‐term datasets, spanning broad geographical and temporal scales, this study contributes to the wider ecological understanding of animals with extensive latitudinal distributions, highlighting intra‐species variance and the need for region‐specific conservation and management.

## Graphical abstract

Species distributed across broad latitudinal gradients often face diverse environmental pressures, yet how these shape intraspecific behavioural variation remains less known. Using long‐term passive acoustic monitoring data of harbour porpoises, from six regions within their Northeast Atlantic latitudinal distribution we found that their foraging behaviour varied with local environmental conditions, diel cycles, and seasons. These results suggest that behavioural flexibility may exist in wide‐ranging species, highlighting the importance of accounting for regional variation in conservation planning.

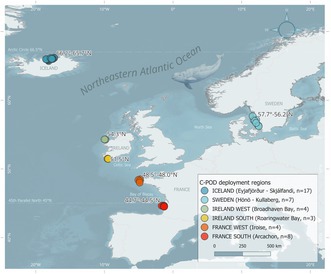

## 1 Introduction

Latitude, the north–south position on the surface of the Earth, is an important factor influencing species diversity and large‐scale patterns in ecology and biogeography. Latitude plays a key role in shaping ecosystems, with the paradigm that species richness increases closer to the equator than to the poles (e.g., Hillebrand 2004) being one of the most widely debated biological patterns (e.g., Chaudhary et al. 2016). How latitude impacts the ecology of species with a wide latitudinal distribution is less known. Indirect or direct latitude‐dependent effects on behaviour and ecology have been found, where factors such as water temperature, daylength and diel phase over latitudinal gradients have effects on mammals (Blix 2016; Daan and Aschoff 1975). For cetaceans, over larger geographical scales, light levels have shown to influence distribution and behaviour of short finned pilot whales (*Globicephala macrorhynchus*) (Owen et al. 2019). Therefore, it is reasonable that cetacean species with a distribution covering a wide range of latitudes, have adapted their ecology to a variety of environmental conditions, and the varying prey species that inhabit those.

Harbour porpoises (*Phocoena phocoena*) are a small widely distributed cetacean species inhabiting primarily coastal and continental shelf waters in the northern hemisphere. In the NE Atlantic, their range extends from the temperate waters northwest off the African continent in the south, to the cold, nutrient‐rich seas of northern Norway, westwards to Greenland, and into the Baltic Sea in the east (Hammond et al. 2013). Strong oceanographic barriers to gene flow have led to three subspecies recognised in the North Atlantic: *P.p. relicta* in the Black Sea, *P.p. meridionalis* in the Iberian‐Mauritania area and *P.p.phocoena* in the North Atlantic (Autenrieth et al. 2024; Fontaine et al. 2007). Within the North Atlantic subspecies, populations differ both genetically and morphologically (Celemín et al. 2023; Fontaine et al. 2007; Galatius et al. 2012). Given the genetic diversity throughout their distribution, harbour porpoises may also differ in ecological aspects.

Regional studies have shown variability in ecology and behaviour of harbour porpoises. In Scandinavian waters, porpoises from the three genetically distinct populations in the Skagerrak, Belt Sea, and Baltic Sea basins (Celemín et al. 2023; Wiemann et al. 2010) exhibit site fidelity during breeding, while moving between neighbouring basins at other times of the year (Sveegaard et al. 2011). In contrast, telemetry studies in Greenland waters show harbour porpoises annually moving over large distances, inhabiting pelagic waters over 2500 m deep, and diving deeper than 400 m (Nielsen et al. 2018). Also, populations exhibit variations in skull morphology, suggesting potential evolutionary adaptations in hunting strategies or prey preferences across regions (Galatius et al. 2012; Galatius and Gol'din 2011). Within the Black Sea, seasonal and diel echolocation patterns of the local sub‐species of harbour porpoises vary across the basin, where prey species and behaviour appear to be the main drivers (Ivanchikova et al. 2024). Regional studies have found that the effects of variables such as tides, and lunar cycles on foraging have varied (Osiecka et al. 2020; Stedt et al. 2023; Todd et al. 2025; Zein et al. 2019). These findings indicate the complexity of harbour porpoise ecology and population structure, which may be shaped by large‐scale environmental factors (Celemín et al. 2023).

The distributional range of harbour porpoises introduces pronounced environmental variability. At higher latitudes, seasonal shifts in daylength and water temperature are extreme: winters feature short days, long nights, and cold surface water, and summers experience up to continuous daylight. Lower latitudes are characterised by more stable environmental conditions year‐round. Salinity levels vary considerably between ocean basins, and together with increased primary production, they are important drivers for several marine mammal species migrations northwards during summer to productive feeding grounds (Gibson et al. 2006).

With the vast distribution of harbour porpoises, from the tropics of West Africa at 14.1° N (Van Waerebeek and Perrin 2007), to well above the Arctic Circle at 66.5° N, it is reasonable to assume they have adapted their ecology to living under the diverse set of conditions that their marine environments provide. For instance, harbour porpoises inhabiting colder waters likely face heightened metabolic demands to maintain body temperature, requiring increased food intake to sustain energy balance (Rojano‐Doñate et al. 2018). As a result, they are sensitive to environmental disturbances, both primary disturbances that affect their ability to forage, such as noise pollution masking their echolocation (Hermannsen et al. 2025), and secondary disturbances influencing prey availability, such as fisheries, environmental pollutants, and habitat degradation.

Nocturnal foraging, increased foraging activity during predominantly darker hours, has been described in harbour porpoises in several areas (Carlström 2005; Ivanchikova et al. 2024; Zein et al. 2019) and is suggested to be linked to zooplankton diel vertical migrations, influencing the movement of pelagic prey fish species such as herring ( *Clupea harengus* ) and sprat ( *Sprattus sprattus* ) (Cardinale et al. 2003; Nilsson et al. 2003). Other prey species, such as sand eel (*Ammodytus marinus*) display different diel patterns, where they burrow during nighttime and feed more during daytime (van der Kooij et al. 2008). Therefore, harbour porpoise foraging strategies and geographical variations of these, may be influenced by spatiotemporal patterns and interactions between prey and environmental conditions.

Understanding the behavioural and ecological patterns of a species, and how these vary between populations, is essential for effective conservation, management, monitoring, and for legislative frameworks such as OSPAR, ASCOBANS, and ACCOBAMS, which coordinate small cetacean conservation across borders. Regional baseline foraging rates, although a metric of foraging activity, rather than consumption metric, could reflect the activity expenditure to maintain energy balance, and could therefore be valuable indicators of population health and viability, as changes in top predator foraging may signal broader ecosystem shifts. However, spatial and temporal variation in harbour porpoise foraging rates remains poorly understood. Establishing such baselines is important for assessing the regional impacts of anthropogenic pressures and environmental change. Disturbances may affect foraging success and, consequently, population health.

Passive acoustic monitoring devices such as C‐PODs (Cetacean POrpoise Detectors, Chelonia Ltd.) are widely used to study harbour porpoises (Carlström 2005; Nuuttila et al. 2018; Pirotta et al. 2014; Schaffeld et al. 2016; Zein et al. 2019). C‐PODs are click detectors, recording information on the acoustic signals of high‐frequency echolocation clicks of odontocetes, and may collect data for months continuously. The recorded clicks may be separated into click types, using the time between the clicks, Inter‐Click‐Interval (ICI). Click trains with ICIs below 10 ms have been used to indicate prey capture attempts (foraging buzzes) (Carlström 2005; Pirotta et al. 2014). Therefore, using the extensive and long‐term datasets collected with these devices, it is possible to identify spatiotemporal foraging hot‐spots, and explore environmental factors that influence foraging.

This study investigates foraging behaviour of harbour porpoises across latitudes within their NE Atlantic distributional range. By utilising C‐POD data from long‐term datasets from a broad latitudinal range across Iceland, Sweden, Ireland, and France, we address two primary research questions: (1) What are the temporal patterns of foraging rates among harbour porpoises in different regions, on a daily and seasonal scale? And (2), how do large‐scale latitude‐related environmental variables relate to foraging behaviour in the regions?

## 2 Methods

**FIGURE 1:**
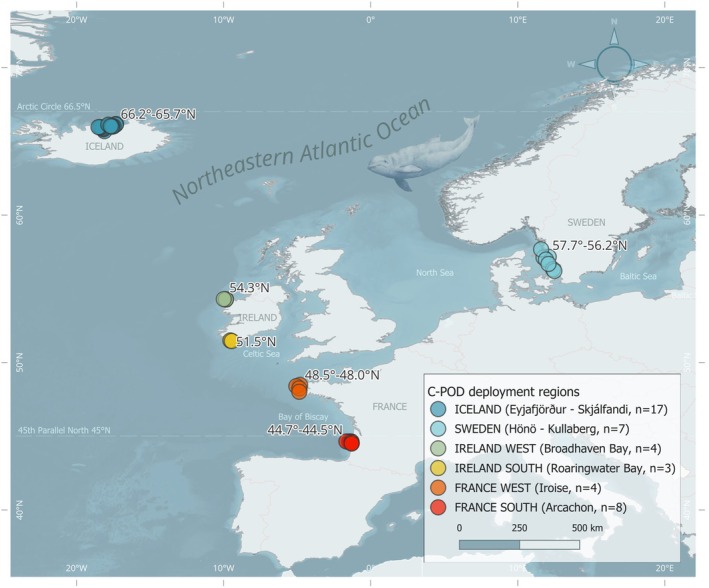
Geographical distribution of deployment regions and sites of passive acoustic recording devices (C‐PODs) analysed for harbour porpoise ( *Phocoena phocoena* ) foraging behaviour. GeoData on bathymetry from EMODnet (European Marine Observation and Data Network 2024) and of country borders from EGM (EuroGlobalMap 2024).

Acoustic data were collected between 2009 and 2023, using C‐PODs deployed coastally across geographic regions within the distribution range of the harbour porpoise in the NE Atlantic. The datasets cover 43 deployment sites across four countries (Figure 1), collected by different projects and research institutions (Table A1). Data from several C‐POD deployment sites within a region were pooled, to represent the acoustic behaviour of harbour porpoises in the broader region, and to minimise variability from small‐scale habitat preferences or events. This resulted in six deployment regions (hereafter referred to as *Iceland*, *Sweden*, *Ireland West, Ireland South*, *France West* and *France South*), representing different latitudes based on the geographical separation from the nearest other region.

From each deployment region, the number of C‐POD deployment sites varied between four and 17, and data from a region were collected for at least one continuous year, and up to seven. The latitudinal range represented by the regions together, range from 44.54° N in France to 66.2° N in Iceland. A summary of deployment sites and regions, and the data collected from these are presented in Table A1. Deployments in Iceland, Sweden and Ireland had mooring designs where C‐PODs were fixed on an anchored mooring line, 2‐5 m above the seabed, with either an acoustic release system, fully submerged riggs that were trawled, or with a surface marker buoy. In France West and South, the deployments were made through a project where different mooring designs for C‐PODs were tested, therefore, the mooring designs in France varied (Samaran and Corman 2015). The seabed at the deployment sites covered mainly sand or muddy bottom substrates or reefs, at depths between 16 and 100 m.

The raw data, the CP1 files from the C‐PODs, were processed using the software CPOD.exe (Version 2.044; Chelonia Ltd.) to produce CP3 files, which includes the detected clicks. The KERNO classifier was used to filter clicks of interest for analysis, where click trains classified as ‘NBHF’ (narrowband high frequency, indicative of harbour porpoise echolocation clicks) with ‘Hi’ (high) and ‘Mod’ (moderate) quality were selected, and click details were exported for further analysis. All statistical analyses were carried out using R (Version 4.4.2, R Core Team 2024).

**FIGURE A1:**
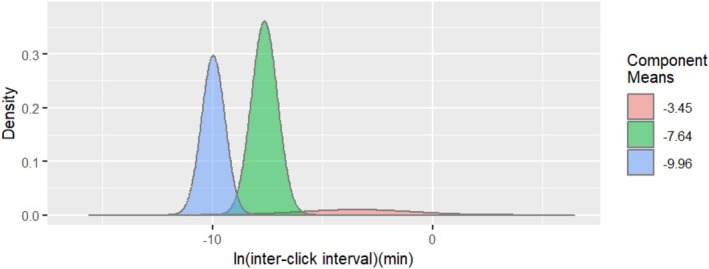
Graph showing an example of the inter‐click‐interval (ICI) distribution of components, from the Gaussian Mixture Model used to classify echolocation clicks of harbour porpoises into click behaviour types. The first peak from the left, with a component mean of −9.96, contains clicks in the dataset with an ICI corresponding to foraging buzzes of harbour porpoises (ICI < 10 ms). The second component peak parallels to ICIs of regular echolocation clicks, and the third wide low peak mainly corresponds to the time between click trains or encounters. Based on their component mean category, the clicks were categorised into either buzz click, regular click or other. This method to classify foraging clicks using the Gaussian mixture model approach was developed by (Pirotta et al. 2014). The data in the graph are from the Eyjafjörður dataset, part of the Iceland region in the present study.

We followed the methods to identify foraging events described in Pirotta et al. 2014, where Gaussian mixture models are employed to classify click types based on ICIs. In their methods, the distribution of ICIs is examined, logarithmic transformation is applied to normalise the data and thereafter, the normalmixEM function from the *mixtools* package (Benaglia et al. 2010) is used to fit the model, to identify the components/distributions of ICIs in the data (K), corresponding to different click behaviours—*buzzes* (click trains with an ICI < 10 ms), *regular clicks* and *others* (largely the time between click trains or encounters) (Figure A1). Click types were assigned to clicks based on the highest posterior probabilities from the mixture model. The data of the clicks were summarised per hour of deployment. Hereafter, the resulting data of buzz clicks, which includes successful and unsuccessful foraging attempts, will be referred to as *foraging* or *foraging behaviour*.

The proportion of time spent foraging was calculated as a ratio of buzz clicks to the total number of clicks detected per hour and used as a measure of foraging rate. The mean hourly foraging rate was visualised as heatmaps per hour across the year per region, overlayed by the local time of sunrise, sunset, dawn and dusk, to highlight potential nocturnal or diurnal foraging patterns. To explore the variability in diel foraging across regions apparent in the heatmaps, foraging events were aggregated by week and diel period (*day* or *night*), then a contrast index was calculated (night − day)/(night + day). We fitted separate linear models for each region with the contrast index as the response, and week as a categorical predictor, omitting the intercept to test deviations from a neutral (0) baseline (even distribution between diurnal/nocturnal foraging). Because we want to study how light levels influence shifts in foraging strategies, we refer to nocturnal or diurnal foraging as a higher proportion of either daytime or nighttime foraging activity. To compare foraging rate across regions, the hourly data were averaged per week, plotted over the year, and visualised in a graph. The R packages ‘*ggplot2*’ (Wickham 2016), ‘*tidyverse’* (Wickham et al. 2019) and ‘*patchwork’* (Pedersen 2025) were used for visualisation, graphs and figures with the colour palette *‘Zissou1’* from the *‘wesanderson’* package (Ram and Wickham 2014).

The effects of latitude related variables on foraging were investigated by Generalised Additive Models (GAMs), one per deployment region, using the ‘*mgcv’* package (Wood 2017). Presence or absence of foraging behaviour per hour was used as the binomial response variable, preventing model overfitting. A binomial distribution and logit link function was used to investigate the relationship between the presence of harbour porpoise foraging (buzzes), and explanatory variables of water temperature, salinity, chlorophyll a, diel phase, and daylength. Diel phase is categorical, while all other explanatory variables are smooth continuous. Data on water temperature, salinity, and chlorophyll a, were retrieved from the Copernicus database, with a spatial resolution of 4 km or otherwise as close to that as available (E.U. Copernicus Marine Service Information). The data from each deployment region were obtained per day and averaged per week, from the first day of data collection in the region until the last, so that the environmental data corresponded to the acoustic data collected. Thereafter, the weekly average was assigned to each hour of porpoise data. For water temperature, both sea surface and bottom temperatures were used, and their average was used to represent the region. If only one was available, that value was used. This approach accounted for gaps in the water temperature datasets, as not all regions had consistent sea surface or bottom temperature data across all time periods of their deployments. Diel phase and daylength associated to each hour of C‐POD data were calculated in several steps. Firstly, the dataset was complemented with derived temporal variables, including hour, Julian day, month, and year, using the *‘lubridate’* package (Grolemund and Wickham 2011). Secondly, the onset of dawn, sunrise, dusk and sunset were calculated per area and day using the package *‘suntools'* (Bivand et al. 2024), and each hour was assigned a diel period (dawn, day, dusk, night). Thirdly, the daylength variable was calculated by subtracting the time of sunset with the time of sunrise. To visualise the transformed fitted relationship between the response and explanatory variables from the GAMs, the ‘*itsadug’* package (van Rij et al. 2022), with plot‐smooth functions, were used.

Collinearity between the environmental variables was tested for every region, and no correlation over 0.7 was found. The ranges of the explanatory variables included in the models varies considerably along latitudes and over seasons, resulting in each region having very different upper and lower limits. By performing full models, and not applying any model selection procedures, biases introduced by stepwise procedures were avoided (Morrissey and Ruxton 2018; Whittingham et al. 2006). Therefore, we do not expect our models to be the ‘best model’ for explaining foraging behaviour in the study areas, but instead to investigate how these large‐scale variables relate to foraging in different regions.

## 3 Results

A total of 126,310 harbour porpoise positive hours (approximately 14.4 years), with a total of 132,171,585 individual porpoise clicks, were analysed to identify buzzes, indicative of foraging behaviour. Of these clicks, 39,754,047 were from Iceland, 86,408,621 from Sweden, 4,563,744 from Ireland West, 864,034 from Ireland South, 40,292 from France West, and 540,847 from France South.

**FIGURE 2:**
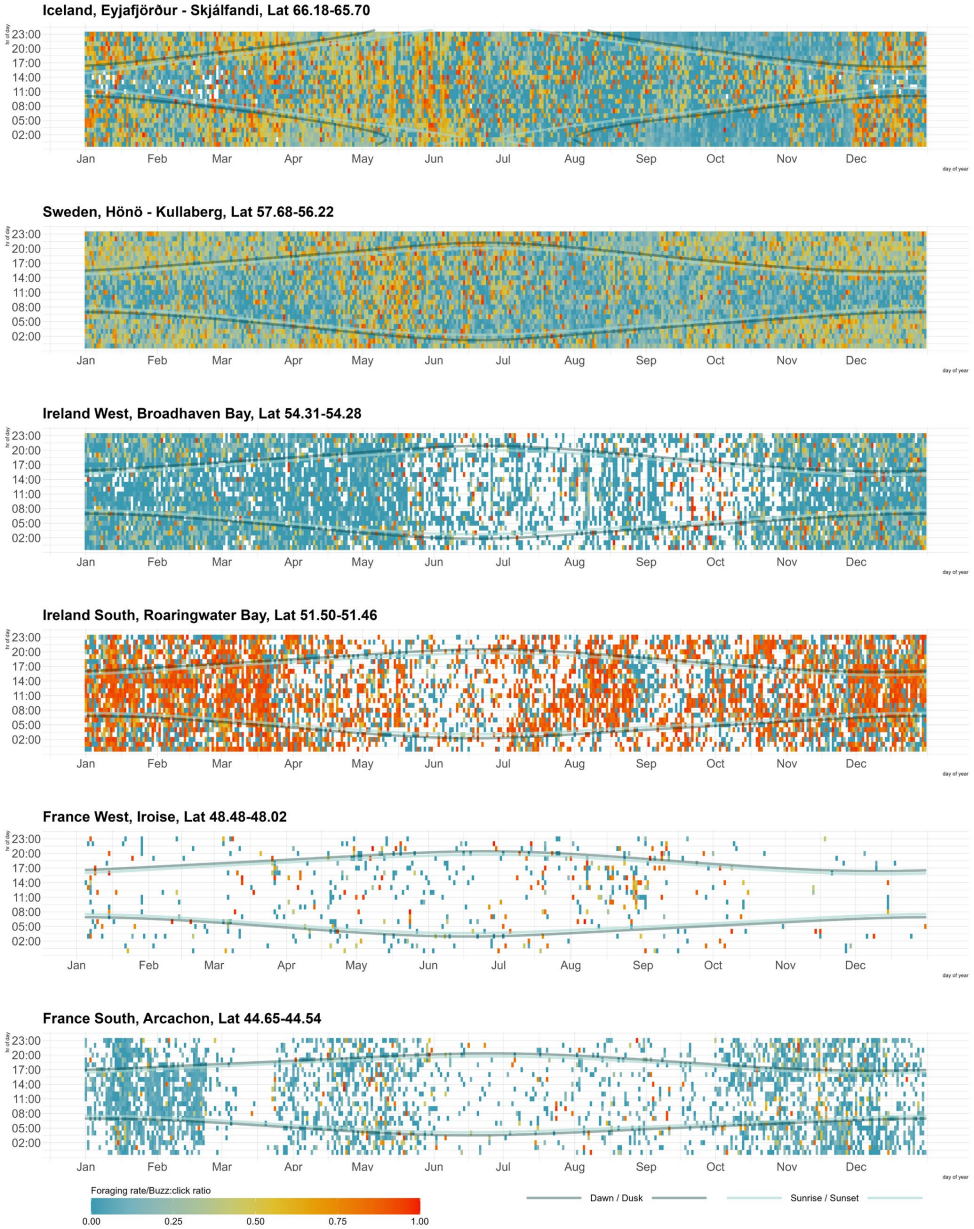
Temporal distribution of foraging rate of harbour porpoises in six regions within their NE Atlantic distribution, with the northernmost area on top, to southernmost at the bottom. The average foraging rate is shown on a blue‐red scale, per hour, over the year, based on accumulated long‐term C‐POD data. Red coloration shows that a high percentage of that hour was spent foraging, whereas blue shows a lower percentage of foraging clicks. White coloration shows absence of echolocation clicks.

**FIGURE 3:**
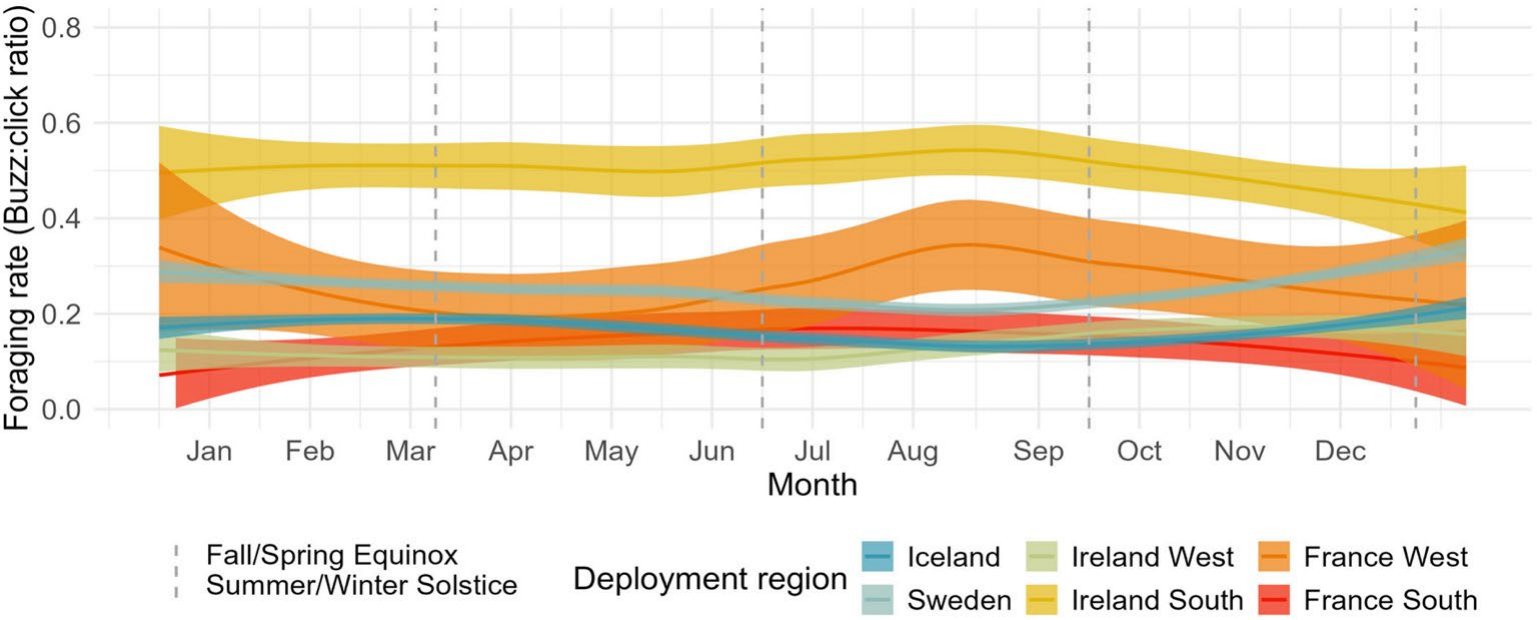
The weekly average foraging rate per deployment region, over seasons, overlayed with the dates of equinox and solstices. The foraging rate is the percentage of echolocation clicks that are foraging attempts. The data are based on long‐term datasets of echolocation clicks, from multiple years and sites within the NE Atlantic range of harbour porpoises, collected using click detectors, C‐PODs.

The visualisations of the temporal foraging rate, shows substantial variations in how and when harbour porpoises forage across areas (Figures 2 and 3). The heatmaps show that in the northernmost regions, Iceland, Sweden and Ireland West, hourly foraging rate for a substantial part of the year, either increases or decreases by the onset of sunrise or sunset, while it does not in the southernmost (Figure 2), also supported by the linear models (Table A2). Predominantly nocturnal foraging is seen in these northern regions, with daytime foraging in Sweden and Iceland during spring and early summer. In Iceland primarily diurnal foraging is observed from August through November (Figure 2 and Table A2). In both Ireland West and Ireland South echolocation clicks are less frequent during summer months, but the foraging rate in the latter is high throughout day and season. The acoustic presence of harbour porpoises in France West is scattered throughout the year, and in France South it is higher from April to June and from October to March. In the linear models, the number of weeks with a significant relationship, or strong trend, between foraging and diel period varied by region, with a latitudinal trend—the northernmost region (Iceland) showed the highest number of significant weeks, followed by Sweden, Ireland West, Ireland South, France West and the southernmost France South, which showed the fewest (Table A2). This pattern suggests stronger diel effect of foraging activity at higher latitudes.

The graph of the weekly average foraging rate shows variety in foraging rate curvature across regions over the year (Figure 3). The highest foraging rate is seen in Ireland South, where harbour porpoises use about 50% of their echolocation clicks to forage through most of the year, with a decrease in late December (Figure 3). In France West, foraging rate peaks at nearly 37% in January and in mid‐August, with troughs around 20%. In France South, the peak is at around 20% in July, with the lowest rate recorded in late December/early January. The foraging rate in Ireland West was consistent through seasons at around 12%, with a soft peak in November at 18%. There is a striking similarity in the curvature between Iceland and Sweden, where the seasonal foraging rate pattern is close to identical, only that harbour porpoises in Sweden appear to feed at a stable 12% higher rate compared to Icelandic porpoises throughout the seasons.

The GAM output shows that the effect of diel phase on foraging behaviour, varied between areas (Table 1), with more prominent effects in the northernmost areas (Figure 2). In both Iceland, Sweden and Ireland West, all diel phases showed significance for foraging behaviour. In Ireland South, there are significant effects of night, dusk and day. No significant effect of diel phase was found for France West, but in France South, there was an increase in foraging during day (Table 1).

**TABLE 1:** Model summaries for the Generalised Additive Models, GAMs, for the six deployment regions in the study, located within the NE Atlantic distribution of harbour porpoises. In the models, the effect of latitude‐related explanatory variables was tested on the binomial foraging behaviour of harbour porpoises.

Explanatory variables	Iceland		Sweden		Ireland West		Ireland South		France West		France South	
	*p*	Effect size	*p*	Effect size	*p*	Effect size	*p*	Effect size	*p*	Effect size	*p*	Effect size
**Parametric coefficients**												
Diel period (relative to Day)	**< 0.001**	1.69	**< 0.001**	1.76	**< 0.001**	−0.30	**< 0.001**	0.97	0.85	−0.02	**< 0.001**	0.98
Dusk	**0.002**	0.21	**< 0.001**	1.03	**0.04**	−0.29	0.75	0.06	0.35	−0.52	0.79	0.08
Dawn	**< 0.001**	0.22	**< 0.001**	0.39	**< 0.001**	0.45	**0.02**	−0.36	0.41	0.59	0.82	0.06
Night	**< 0.001**	0.21	**< 0.001**	0.95	**< 0.001**	0.50	**0.002**	−0.18	0.77	0.06	0.12	0.15
**Smooth terms**												
Water temperature	**0.003**	6.10	**< 0.001**	8.83	**< 0.001**	5.28	**< 0.001**	8.43	**< 0.001**	8.39	**< 0.001**	7.54
Salinity	**< 0.001**	8.31	**< 0.001**	8.84	**< 0.001**	8.34	**< 0.001**	5.58	0.16	2.97	**< 0.001**	8.12
Chlorophyll a	**< 0.001**	5.94	**< 0.001**	8.77	**0.023**	4.29	**< 0.001**	7.44	*0.07*	7.25	**< 0.001**	8.79
Daylength	**< 0.001**	7.95	**< 0.001**	4.43	**< 0.001**	7.88	**< 0.001**	8.00	0.51	0.00	**< 0.001**	7.34
Num. obs.	51,116		46,445		10,583		6822		503		2970	
*R* ^2^ adj.	0.012		0.038		0.036		0.037		0.079		0.183	
AIC	42,019		30,535		14,312		8128		681		3078	

*Note:* Significant interactions are indicated by *p*‐value < 0.05, and bold text, and strong trends (*p*‐value between 0.05 and 0.10) with cursive text. Effect size is derived from F‐statistic for smooth terms, and *t* value for fitted coefficients.

**FIGURE 4:**
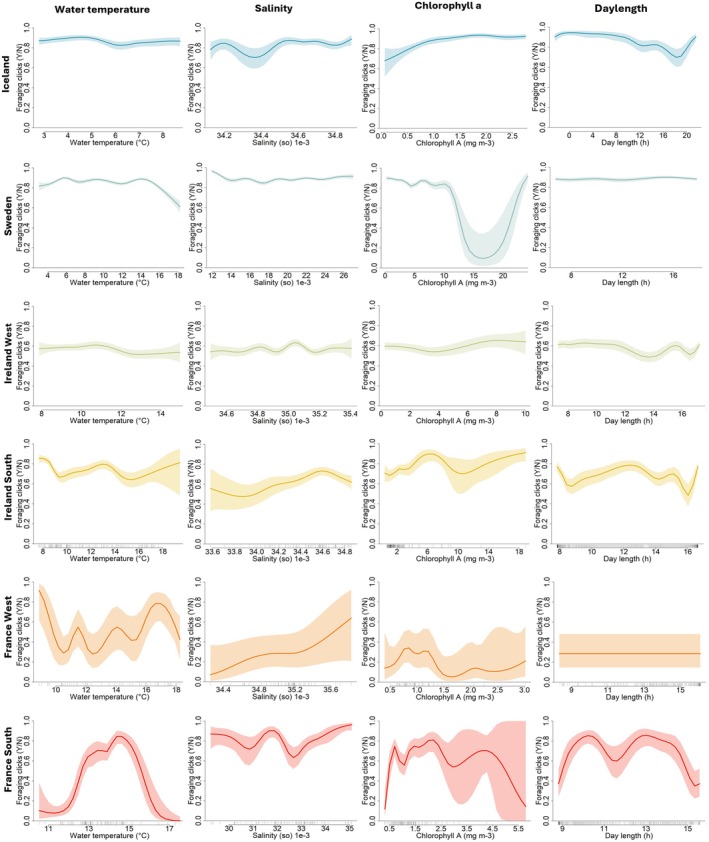
Output from the six Generalised Additive Models, visualising the smooth relationships between the explanatory variables on harbour porpoise foraging behaviour. The fitted relationship to water temperature, sea water salinity, chlorophyll a, and daylength is illustrated left to right, and the results from the six regions within the NE Atlantic distributional area of harbour porpoises, are illustrated top to bottom, with the northernmost region on top. Shaded areas represent associated 95% confidence intervals.

The smooth terms included in the GAMs shows varying effects on foraging behaviour (Figure 4 and Table 1). The effect of water temperature is significant, with relatively high effect sizes across all regions. The highly varying response across regions, shows that temperature plays a complex, non‐linear relationship with foraging. The smooth terms for salinity, chlorophyll a, and daylength, has significant effects in all regions, apart from France West, also with effect sizes showing complex relationship to foraging. In France West, the response is flat, with a constant variation across the daylength range. Overall, our models show that these large‐scale environmental variables play important roles in the relationship with foraging behaviour in the regions.

## 4 Discussion

This study highlights the diversity in ecological adaptations within a widely distributed species. The strong differences in seasonal and diurnal foraging patterns observed, indicate that harbour porpoise ecology varies across geographic regions. Nocturnal foraging, a higher proportion of foraging activity during darker hours, is seen in the northernmost regions, aligning with previous findings (Carlström 2005; Osiecka et al. 2020; Schaffeld et al. 2016; Zein et al. 2019). However, the opposite diel trend—diurnal foraging—is observed in Iceland between August and November. Thereafter, harbour porpoises switch foraging strategy and forage more intensely during hours of darkness (Figure 2). Both the nocturnal and diurnal foraging patterns associate with the onset of sunrise or sunset, as shown by the foraging rate either increasing or decreasing at those times.

Interestingly, none of the three southernmost deployment regions—Ireland South, or the two in France, showed an increase in nocturnal foraging (Figure 2, Table 1 and Table A2). These findings challenge previous findings that harbour porpoises primarily are nocturnal foragers. Instead, our results suggest that foraging behaviour is more related to diel phase in the harbour porpoises northerly distributional range in NE Atlantic, and therefore that a non‐even distribution of foraging between diel phases, nocturnal and diurnal foraging, is more prominent northwards. As most research on harbour porpoises has been conducted in their northern distributional range in NE Atlantic, it is possible that this may bias our knowledge on the species. In the Atlantic, harbour porpoises range as far south as the tropical waters of Senegal. This most southernmost area along the northwestern African coast remains significantly understudied (Boisseau et al. 2007; Van Waerebeek and Perrin 2007). Even more broadly, local studies of harbour porpoises in the Pacific Ocean and NW Atlantic have shown seasonal patterns in acoustic detections (Dracott et al. 2022; Hall 2011; Trabue et al. 2025) and foraging (Holdman et al. 2025; Maeda et al. 2021), showing comparable foraging rates as in the present study (Holdman et al. 2025). But large scale seasonal and diurnal foraging rates, in these basins and beyond, would be needed for a comprehensive overview of harbour porpoise ecology.

In both Iceland and Sweden, the predominant nocturnal foraging in winter and spring, is disrupted in early summer, coinciding with the calving and weaning time period (Bjørge and Tolley 2009; Börjesson and Read 2003; Figure 2). During this period, foraging rate increases substantially, and throughout the day, suggesting a heightened energetic demand during critical life history stages. The variability in how harbour porpoises feed through seasons, highlights that short‐term behavioural data from individuals should only carefully be extrapolated to represent longer‐term (Wisniewska et al. 2016). In fact, or study shows that harbour porpoises may allocate their foraging efforts non‐evenly throughout diel phases and seasons, which refutes earlier premises that they need to forage continuously to meet energetic demands (Wisniewska et al. 2016).

Contrary to the increased day‐round foraging effort during early summer in northern regions, there is instead a notable decrease in harbour porpoise detections in both Ireland West and Ireland South throughout summer months and into fall. Although the movements of these harbour porpoises remain unknown, the similarity in their seasonal absence in both Irish regions raises questions about whether they move to shared breeding and calving grounds or occupy distinct areas during that period. Tracking studies of harbour porpoises from Greenland in May showed that individuals travel exceptionally long distances, moving from west of Greenland (64° N, 60° W), across the continental shelf and offshore waters near 55° N, 25° W (Nielsen et al. 2018). This area is relatively close to the Ireland West deployment region in the present study, and the findings indicate that long‐distance movements are possible for the species. Other studies identified the German Wadden Sea, as an important foraging area, particularly before and during the breeding season (Zein et al. 2019). Further research could help clarify where these animals may move during breeding and calving season.

Although the foraging rates in the present study reflects an activity proxy, rather than a consumption metric, it is still expected that harbour porpoises in colder northern waters would exhibit higher foraging rates to meet increased energetic demands. Our results do not support this hypothesis, as the northernmost regions in the study do not show elevated foraging rates compared to regions further south. In contrast, the Ireland South region—which includes the designated Special Area of Conservation (SAC) Roaringwater Bay—exhibited the highest foraging rate of all surveyed areas. Harbour porpoises in this area foraged intensively across both diurnal and seasonal scales (Figures 2 and 3). However, the similarity in seasonal foraging rate patterns between Iceland and Sweden, where their curvatures are close to identical (Figure 3), show that harbour porpoises in these two regions might have similar adaptations to handle extreme shifts in their environment. Their observed peaks during winter align with increased energy demands during colder months, mirroring described seasonal physiological changes, such as increased blubber thickness to reduce heat loss (Rojano‐Doñate et al. 2018). Future studies across the distribution range of harbour porpoises, combining passive acoustic monitoring data with data on blubber thickness over seasons, would provide a clearer picture on energy expenditure across seasons. The variabilities in foraging rates across regions both highlight the temporal ecological significance of regions, and possibly also reflect prey availability and/or nutritional status. Since the seasonal foraging patterns presented here are based on large‐scale, multi‐year, compiled datasets, our results may represent foraging strategies used by porpoises in different regions to meet their energetic demands. As such, these seasonal foraging rate baselines could serve as reference points for future studies on harbour porpoise foraging behaviour, especially in the context of warming oceans and anthropogenic impacts.

Absence of harbour porpoise detections or foraging events, observed as scatter or acoustic silence in the heatmaps (Figure 2) were recorded in several deployment regions, and may reflect a range of biological factors, such as a reduced energetic demand, consumption of more energy‐rich prey, or fewer unsuccessful foraging attempts. Harbour porpoises may relocate, areas may serve as transit routes or may only occasionally be used for feeding. Variability in area usage suggests mobile and flexible porpoises, sometimes foraging locally, at other times offshore or along the coast. Harbour porpoises may also use predator avoidance strategies such as decreasing their acoustic activity, or avoiding areas (Samarra et al. 2018; Todd et al. 2020), to lower the risk of detection, as other cetacean species (Castellote et al. 2022). Additionally, so‐called ‘silent dives’ have been described for harbour porpoises during sleep (Wright et al. 2017), a behaviour likely essential given the importance of sleep in mammals (Siegel 2005; Tobler 1995). Therefore, while occurrence of foraging behaviour indicates the ecological importance of an area, a lower foraging rate, should not be interpreted as indicators of insignificant areas.

Our analysis shows that broad‐scale environmental variables, such as water temperature, primary production and salinity, play complex and sometimes contrasting roles in harbour porpoise foraging behaviour across the study regions. Although these variables fluctuate over seasons and often vary more at higher latitudes, they do not vary linearly with latitude, due to geographic and oceanographic influences. For example, salinity in France South is affected by freshwater influx from estuaries, and warmer water temperature in Iceland is influenced by the Gulf Stream. However, the predictability and stability of the underlying factors affecting these large‐scale variables across latitudes, such as seasonality, Earth's axial tilt, and ocean currents, means they may influence foraging strategies, primarily because prey and fish species and populations differ in their tolerance to levels of abiotic factors such as salinity and temperature (dos Santos Schmidt and Kennedy 2023; Marshall and Elliott 1998). Therefore, the complexity of the effects the predictors have on harbour porpoise foraging in regions, might reflect the diverse, and sometimes cumulative effect, they may have on prey and fish species communities, such as migration patterns and spawning (Pörtner and Peck 2010), which foraging strategies of harbour porpoises may directly depend upon. Thus, as environmental conditions shift, and fish species communities alter as a response, harbour porpoise foraging strategies may change as well.

Primary prey for harbour porpoises in the Iceland deployment region, covering the feeding ground Skjálfandi (Charles et al. 2025), are capelin ( *Mallotus villosus* ) and Atlantic cod ( *Gadus morhua* ) (Koponen 2013; Pampoulie et al. 2023; Singh et al. 2024; Víkingsson et al. 2003) and the area constitutes a nursery ground for capelin during summer months, when porpoises also feed on their eggs (Bárðarson 2021; dos Santos Schmidt and Kennedy 2023; Koponen 2013). In Iceland, the foraging relationship with water temperature is relatively stable, but with a slight decrease in foraging at 6°C (Figure 4). Interestingly, this specific temperature corresponds to the maximum temperature for optimal capelin density, and Atlantic cod north of Iceland (dos Santos Schmidt and Kennedy 2023; Righton et al. 2010). In Sweden, foraging peaks at 5°C, 9°C and 14°C (Figure 4), where gadoids and clupeids constitute a substantial part of the diet (Stedt et al. 2025). Gadoids such as cod primarily occupy waters between 2°C and 15°C in the Skagerrak and Kattegatt basin, with optima at around 5°C, similar to clupeids such as herring and sprat (Peltonen et al. 2007; Righton et al. 2010). The optimal temperatures of prey species in both Iceland and Sweden correspond to the peaks in foraging behaviour observed in the GAM outputs (Figure 4). Just as harbour porpoise foraging behaviour peaks in Iceland and Sweden correspond to optimal temperatures for main prey items—peaks in the other regions may correspond to optimal temperatures and abiotic factors for their prey species. Therefore, the complexity in our GAM output may indicate shifts in prey species, as the environmental variables fluctuate with seasons. Additionally, vertical migrations of prey species such as sprat and herring (Blaxter and Parrish 1965; Cardinale et al. 2003; Nilsson et al. 2003), where they move towards the surface at night and shoal in response to shifts in light intensity, may be drivers for the nocturnal foraging described in the present study. Comparable relationships between temporal variations in harbour porpoise activity and prey species ecology have been described in the Black Sea (Ivanchikova et al. 2024), where seasonal and vertical migrations of prey species, such as anchovy (*Engraulis encrasicolus L*) and sprat, were consistent with harbour porpoise activity. This implies that the behaviour of prey species are ecological drivers for seasonal and diel dynamics of harbour porpoises.

It is important to consider that there may be intra‐population variations in how harbour porpoises use an area. Within‐population diet differences between sexes and age groups have been found in Greenland and Sweden (Louis et al. 2022; Stedt et al. 2025). Therefore, variances in spatiotemporal foraging patterns, behaviour, and habitat preferences, that mirror similar differences in diet are possible. Dietary differences within a population may minimise intra‐species competition, aligning with the Ideal Free Distribution theory (Fretwell 1972; Fretwell and Lucas 1969), which proposes that individuals distribute themselves among resource patches to maximise fitness and minimise competition. Therefore, areas may serve different roles in meeting individual energy requirements, depending on factors such as age, sex, reproductive status, season and time of day. With data collection using C‐PODs, acoustic activity from all porpoises present is captured, and analysed together during processing. Thus, while current analytical methods keep possible intra‐population patterns hidden, future efforts to analyse echolocation signals further in‐depth, such as separating clicks from adults and calves, may provide greater insight into the significance of areas. Additionally, further dietary studies, that compare age groups and sexes across harbour porpoises' distributional range, would greatly enhance our understanding of their foraging ecology. Such efforts would contextualise foraging behaviours, seasonal and diurnal patterns, and the influence of environment, identified in the present study.

While our findings underscore the ecological diversity of harbour porpoises across their NE Atlantic range, the challenge now lies in understanding what this heterogeneity means for the species as a whole. This ecological diversity may suggest that harbour porpoises are more adaptive than previously understood—a potential strength that implies populations could adjust to environmental changes if given the opportunity. Alternatively, the observed variation across regions might indicate a high degree of specialisation and local dependency, which could be a vulnerability. In that case, tailored conservation strategies, particularly within protected areas, may be essential to support regionally specific ecological needs. This uncertainty has implications for understanding how flexible or vulnerable different populations may be to local threats, such as bycatch, diseases, and predation (Neimanis et al. 2022). To fully understand the ecological diversity of harbour porpoises, several key knowledge gaps remain, as highlighted in this study: Where do harbour porpoises go during their seasonal absences? To what extent—and why—are porpoises present but acoustically silent? And what are the principle drivers of regional differences in foraging rates?

When consistent ecological data collection methods are applied for extended periods and broad geographical scales, they can significantly enhance conservation efforts and our understanding of ecosystem dynamics. Long‐term datasets generated through C‐POD deployments played a key role in advancing conservation efforts for regional harbour porpoise populations, designating the Baltic Proper harbour porpoise ‘Critically Endangered’ (Carlström et al. 2023), by identifying key areas and informing management strategies (Amundin et al. 2022; Carlén et al. 2018). In the present study, we used C‐POD data covering approximately 14.4 years of harbour porpoise positive hours, within their distributional range in the NE Atlantic, with results that provide a better ecological understanding of them as a species. To our knowledge, this is the first study to present a distribution‐wide perspective on harbour porpoise acoustic foraging behaviour, spanning from the Arctic to the subtropical waters. We encourage similar collaborative efforts across more of the harbour porpoise's distribution, as well as to other widely distributed marine mammal species. Our findings demonstrate that long‐term datasets—spanning broad geographical and temporal scales—and cross‐basin collaborations can yield valuable baseline insights into species ecology.

## Author Contributions

**Jasmine Stavenow Jerremalm:** conceptualization (lead), formal analysis (lead), investigation (lead), methodology (lead), project administration (lead), validation (equal), visualization (lead), writing – original draft (lead), writing – review and editing (lead). **Julie Beesau:** data curation (equal), writing – review and editing (equal). **Julia Carlström:** conceptualization (equal), data curation (equal), resources (equal), validation (equal), writing – review and editing (equal). **Pia Eriksson:** conceptualization (equal), data curation (equal), resources (equal), validation (equal), writing – review and editing (equal). **Mark Jessopp:** conceptualization (equal), methodology (equal), resources (equal), supervision (equal), validation (equal), writing – review and editing (equal). **Ailbhe Kavanagh:** conceptualization (equal), methodology (equal), resources (equal), supervision (equal), validation (equal), writing – review and editing (equal). **Olli Loisa:** data curation (equal), writing – review and editing (equal). **Mathilde Michel:** data curation (equal), writing – review and editing (equal). **Kylie Owen:** conceptualization (equal), data curation (equal), resources (equal), validation (equal), writing – review and editing (equal). **Marianne Helene Rasmussen:** data curation (equal), writing – review and editing (equal). **Flore Samaran:** data curation (equal), writing – review and editing (equal). **Nicole Rose Eileen Todd:** data curation (equal), resources (equal), writing – review and editing (equal). **Emer Rogan:** conceptualization (equal), funding acquisition (lead), methodology (equal), resources (equal), supervision (lead), validation (equal), writing – review and editing (equal).

## Funding

J.S.J. is funded by the Sustainable Energy Authority of Ireland under the SEAI Research, Development & Demonstration Funding Programme 2021 (grant number 21/RDD/670).

## Disclosure

Statement on inclusion: Our study brings together authors from different countries, and includes scientists based in the countries where the data collections were carried out. Whenever relevant, literature published by scientists from the region was cited; efforts were made to consider relevant work published in the local language.

## Conflicts of Interest

The authors declare no conflicts of interest.

## Supporting information


**Data S1:** Supporing information.

**Data S2:** Supporing information.

## Data Availability

Data and code that supports the main findings from this study are uploaded as Data S1. The data sources for the environmental data used in the study, from the E.U. Copernicus Marine Service Information, are provided in the Data sources section.

## Appendix A

**TABLE A1:** Summary of the C‐POD deployment sites and data that was analysed in the present study. Acoustic data were collected using C‐POD (click detectors) at the 43 deployment sites from 2009 to 2023. For the analysis, data from the deployment sites were pooled into six regions, based on their latitude.

Site no	Site ID	Deployment region	Geographical coverage	Latitude	Longitude	Depth (m)	Data from	Data until	Responsible research institution and project
1	SKJAL14	Iceland	Eyjafjörður‐Skjálfandi	66.18	−17.31	92	Aug‐17	Aug‐18	Turku University of Applied Sciences and University of Iceland, as part of the Arctic Climate Predictions: Pathways to Resilient, Sustainable Societies (ARCPATH) project
2	SKJAL18			66.14	−17.33	60	Aug‐17	Aug‐18	
3	SKJAL37			66.13	−17.84	44	Aug‐17	Aug‐18	
4	SKJAL19			66.09	−17.76	36	Aug‐17	Aug‐18	
5	SKJAL91			66.04	−17.67	33	Aug‐17	Aug‐18	
6	SKJAL69			66.04	−17.58	100	Aug‐17	Aug‐18	
7	SKJAL48			66.03	−17.48	70	Aug‐17	Aug‐18	
8	SKJAL74			66.00	−17.59	15	Aug‐17	Aug‐18	
9	EYJA92			65.97	−18.50	20	Aug‐17	Aug‐18	
10	EYJA70			65.97	−18.41	35	Aug‐17	Aug‐18	
11	EYJA49			65.96	−18.31	62	Aug‐17	Aug‐18	
12	EYJA28			65.95	−18.21	27	Aug‐17	Aug‐18	
13	EYJA33			65.91	−18.22	75	Aug‐17	Aug‐18	
14	EYJA36			65.87	−18.24	92	Aug‐17	Aug‐18	
15	EYJA13			65.87	−18.14	58	Aug‐17	Aug‐18	
16	EYJA17			65.83	−18.16	93	Aug‐17	Aug‐18	
17	EYJA1			65.70	−18.11	38	Aug‐17	Aug‐18	
18	BALGO1	Sweden	Hönö‐Kullaberg	57.17	12.11	16	May‐19	Apr‐23	Swedish Museum of Natural History, Sweden as part of the national monitoring of the population assessment. Results are published as an annual Swedish report, to the Swedish Water Management
19	FLAD2			57.13	11.76	23	May‐19	Apr‐23	
20	HONO			57.68	11.59	30	Oct‐22	Sep‐23	
21	LMIDD1			56.99	11.92	27	May‐19	Apr‐23	
22	NVSK4			56.33	12.40	27	Aug‐21	Oct‐22	
23	NVSK6			56.22	12.49	21	May‐19	Apr‐23	
24	STMIDD1			56.69	12.09	25	May‐19	Apr‐23	
25	LS1	Ireland West	Broadhaven bay	54.29	−9.85	14	Apr‐09	Oct‐15	University College Cork, Ireland
26	LS2			54.29	−9.84	18	Jul‐09	Jul‐17	
27	LS3			54.28	−9.85	16	Apr‐09	Jul‐16	
28	LS4			54.31	−9.99	37	Mar‐10	Jul‐17	
29	CALF	Ireland South	Roaringwater bay	51.48	−9.50	20	Nov‐20	May‐23	
30	LONG			51.50	−9.54	18	Apr‐21	May‐23	
31	SHERKIN			51.46	−9.44	23	Nov‐20	May‐23	
32	LINIOU	France West	Iroise	48.49	−4.80	30	Jul‐12	Sep‐13	Ensta Bretagne University, France, as part of the MARSAC and LISEA project
33	MAREE			48.40	−5.05	43	Dec‐13	Apr‐14	
34	LAVANDREE			48.25	−4.85	38	Apr‐13	Jul‐13	
35	AWAC			48.02	−4.85	16	Oct‐12	Dec‐13	
36	LISEA1	France South	Arcachon	44.65	−1.45	50	Nov‐15	Nov‐16	
37	LISEA2			44.64	−1.32	30	Nov‐15	Nov‐16	
38	LISEA3			44.54	−1.29	20	Mar‐16	Dec‐16	
39	LISEA4			44.60	−1.45	50	Nov‐15	Nov‐16	
40	MRC1			44.65	−1.65	50	Jan‐13	Dec‐13	
41	MRC2			44.63	−1.30	30	Mar‐13	Sep‐13	
42	MRC3			44.54	−1.29	20	May‐13	May‐14	
43	MSC4			44.51	−1.29	30	Jun‐13	Mar‐14	

**TABLE A2:** Model summaries for the Generalised Linear Models, GLMs, for the six deployment regions in the study. The effect of day or night was tested on the binomial foraging behaviour of harbour porpoises, per week over the year, to describe how foraging activity is distributed throughout the daylight hours over the year. A statistical significance or trend with a positive effect size corresponds to a primarily nocturnal foraging behaviour, while statistical support with a negative effect size corresponds to predominantly diurnal foraging effort for that week.

Factor (week)	Iceland		Sweden		Ireland West		Ireland South		France West		France South	
	*p*	Effect size	*p*	Effect size	*p*	Effect size	*p*	Effect size	*p*	Effect size	*p*	Effect size
1	**< 0.001**	0.636	*0.076*	0.364	0.133	0.304	0.193	0.273	0.638	−0.2	0.513	0.143
2	**< 0.001**	0.619	0.128	0.304	0.115	0.333	0.193	0.273	0.638	0.2	0.394	0.182
3	**0.002**	0.545	0.128	0.304	0.187	0.273	0.193	0.273	0.294	−0.5	0.394	0.182
4	**0.009**	0.455	0.183	0.273	0.187	0.273	0.288	0.217	*0.037*	1	0.394	0.182
5	**0.022**	0.391	0.183	0.273	0.283	0.217	0.406	0.167	0.294	1	0.67	0.091
6	*0.075*	0.304	0.395	0.167	0.4	0.167	0.406	0.167	0.294	1	0.827	0.048
7	0.119	0.273	0.395	0.167	0.4	0.167	0.524	0.13	0.139	−1	0.683	0.083
8	0.319	0.167	0.514	0.13	0.5	0.143	0.664	0.091	0.139	1	1	0
9	0.603	0.091	0.828	0.043	0.66	0.091	0.832	0.043			1	0
10	0.799	0.043	1	0	1	0	1	0	0.638	0.2		
11	0.799	−0.043	0.828	−0.043	1	0	1	0	*0.07*	−1	0.317	−1
12	0.445	−0.13	0.67	−0.083	0.66	−0.091	0.664	−0.091	0.294	1	*0.058*	−0.6
13	0.203	−0.217	0.514	−0.13	0.5	−0.143	0.524	−0.13	0.16	−0.6	0.513	−0.143
14	*0.075*	−0.304	0.277	−0.217	0.519	−0.13	0.524	−0.13	*0.088*	−0.667	0.491	−0.158
15	**0.038**	−0.364	0.277	−0.217	0.283	−0.217	0.664	−0.091	1	0	0.346	−0.222
16	**0.022**	−0.391	0.128	−0.304	0.187	−0.273	0.288	−0.217	1	0	0.394	−0.182
17	**0.002**	−0.545	*0.076*	−0.364	0.133	−0.304	0.172	−0.3	0.691	−0.143	0.201	−0.273
18	**< 0.001**	−0.636	**0.051**	−0.391	*0.092*	−0.333	*0.082*	−0.364	0.391	0.333	0.394	−0.182
19	**< 0.001**	−0.667	**0.034**	−0.417	**0.028**	−0.455	*0.082*	−0.364	0.458	−0.25	**0.05**	−0.429
20	**< 0.001**	−1	**0.017**	−0.478	**0.028**	−0.455	**0.007**	−0.647	0.544	0.333	0.491	−0.158
21	**< 0.001**	−1	**0.008**	−0.545	**0.028**	−0.455	**0.016**	−0.556	0.691	−0.143	1	0
22	**< 0.001**	−1	**0.008**	−0.545	*0.052*	−0.444	0.308	−0.25	0.294	−1	0.439	−0.2
23	**< 0.001**	−1	**0.008**	−0.545	**0.006**	−0.6	*0.053*	−0.6	0.544	−0.333	0.103	−0.667
24	**< 0.001**	−1	**0.002**	−0.636	**0.028**	−0.571	**0.023**	−0.5	0.139	1	1	0
25	**< 0.001**	−1	**0.002**	−0.636	**0.052**	−0.444	**0.004**	−0.733	0.139	−1	0.317	0.5
26	**< 0.001**	−1	**0.002**	−0.636	**0.039**	−0.5	*0.084*	−0.412	0.638	0.2	0.414	−0.333
27	**< 0.001**	−1	**0.002**	−0.636	**0.01**	−0.625	*0.069*	−0.4	0.16	−0.6	**0.014**	−1
28	**< 0.001**	−1	**0.008**	−0.545	**0.003**	−0.833	**0.03**	−0.455	0.691	0.143	0.564	0.333
29	**< 0.001**	−1	**0.008**	−0.545	**0.03**	−0.636	0.069	−0.4	0.544	−0.167	0.206	−0.4
30	**< 0.001**	−1	**0.017**	−0.478	*0.074*	−0.5	**0.03**	−0.455			0.257	−0.429
31	**< 0.001**	−0.604	**0.026**	−0.455	**0.025**	−0.529	*0.082*	−0.364	*0.088*	−0.667	0.109	−0.429
32	**< 0.001**	−0.636	**0.051**	−0.391	0.133	−0.304	0.137	−0.304	**0.006**	−1	0.18	0.6
33	**0.002**	−0.545	0.128	−0.304	0.187	−0.273	*0.069*	−0.4	**0.011**	−1	0.225	−0.294
34	**0.009**	−0.455	0.128	−0.304	0.115	−0.333	0.193	−0.273	0.391	−0.333	0.317	−0.333
35	**0.038**	−0.364	0.277	−0.217	0.627	−0.111	0.459	−0.176	*0.07*	−0.5	0.781	−0.077
36	0.135	−0.25	0.277	−0.217	*0.098*	−0.368	0.824	−0.048	1	0	0.763	−0.091
37	0.298	−0.182	0.514	−0.13	1	0	1	0	0.139	0.5	0.414	−0.333
38	0.445	−0.13	0.67	−0.083	0.5	−0.143	0.832	−0.043	0.139	1	0.467	−0.176
39	0.618	−0.083	0.67	−0.083	1	0	1	0	0.544	0.333	0.317	−0.333
40	1	0	1	0	0.813	−0.053	0.483	−0.158	0.638	0.2	0.564	0.167
41	0.618	0.083	0.67	0.083	0.645	0.1	0.824	−0.048	0.294	−0.5	0.827	0.048
42	0.298	0.182	0.67	0.083	0.167	0.3	0.677	0.083	0.294	−1	0.67	0.091
43	0.203	0.217	0.514	0.13	0.167	0.3	0.505	0.143			0.513	0.143
44	*0.075*	0.304	0.374	0.182	0.379	0.182	0.266	0.238	0.294	1	0.532	0.13
45	**0.038**	0.364	0.277	0.217	0.283	0.217	0.385	0.182			0.532	0.13
46	**0.022**	0.391	0.202	0.25	0.283	0.217	0.288	0.217	1	0	0.414	0.167
47	**0.002**	0.545	0.128	0.304	0.207	0.25	0.288	0.217			0.414	0.167
48	**0.002**	0.545	*0.076*	0.364	0.133	0.304	0.212	0.25	0.294	1	0.297	0.217
49	**< 0.001**	0.7	*0.076*	0.364	*0.079*	0.364	0.137	0.304	0.139	1	0.18	0.3
50	**< 0.001**	0.7	*0.076*	0.364	*0.079*	0.364	0.137	0.304	0.544	−0.333	0.18	0.3
51	**< 0.001**	0.714	*0.076*	0.364	*0.079*	0.364	0.137	0.304			*0.074*	0.4
52	**< 0.001**	0.714	*0.076*	0.364	0.133	0.304	0.137	0.304			0.201	0.273
53	**< 0.001**	0.714	*0.076*	0.364	0.183	0.333	0.362	0.2	0.294	1	1	0
Num.Obs.	1179		1207		1086		1127		198		808	
*R* ^2^ adj.	0.328		0.08		0.06		0.037		0.098		0.001	
AIC	2930		3378		3069		3209		583		2344	

*Note:* Significant interactions are indicated by *p*‐value < 0.05 and bold text, and strong trends (*p*‐value between 0.05 and 0.10) with cursive font.
